# Characteristics of the pediatric patients diagnosed with SARS-CoV-2 infection in a Romanian children’s hospital: a retrospective study

**DOI:** 10.7717/peerj.11560

**Published:** 2021-06-04

**Authors:** Liana-Cătălina Gavriliu, Carmen Murariu, Vladimir Potop, Radu Spătaru

**Affiliations:** 1 Carol Davila University of Medicine and Farmacy, Bucharest, Romania; 2 “Marie Curie” Emergency Children’s Hospital, Bucharest, Romania; 3 University of Pitesti, Pitesti, Romania; 4 State University of Physical Education and Sport, Chisinau, Moldova

**Keywords:** COVID-19, Clinical and epidemiological characteristics, Symptomatic and asymptomatic patients, Symptoms, Laboratory and radiological characteristics

## Abstract

**Background:**

To date, information on COVID-19 pediatric patients is still sparse. We aimed to highlight the epidemiological and clinical data regarding SARS-CoV-2 infection in children and adolescents to improve the understanding of the disease in this age group and inform physicians during the ongoing COVID-19 pandemic.

**Methods:**

We conducted a retrospective, observational study in “Marie Curie” Emergency Children’s Hospital from Bucharest, Romania. We analyzed clinical and epidemiological characteristics of the patients confirmed with SARS-CoV-2 infection, between April 1, 2020–October 31, 2020.

**Results:**

A total of 172 patients aged 0–18 years were included, 79 (45.93%) female and 93 (54.07%) male patients. 28 (16.28%) patients had co-morbidities (more often identified in asymptomatic group; *p* < 0.0001). 47 (27.32%) had exposure to an identified source. 30 (17.44%) patients were asymptomatic; 142 (85.56%) had mild or moderate disease. The most frequent symptoms were: pyrexia (78.87%), digestive symptoms (50%), cough (40.14%). Chest X-ray was performed in 50 patients and it was abnormal in half of them, all being symptomatic. About 2/3 of the evaluated patients had normal leukocytes. The most common hematological change was lymphopenia; monocytes tended to be higher in symptomatic patients. About 40% of the patients were admitted; none required admission to ICU. No significant differences were found between symptomatic and asymptomatic patients regarding gender, age distribution, and exposure to a source.

**Conclusions:**

All the patients had asymptomatic, mild or moderate disease. Patients with comorbidities, classically considered high risk patients, presented the same pattern of disease.

## Introduction

Global spreading of the new coronavirus disease (COVID-19), caused 19,44,888,869 cases confirmed all over the world, resulting in up to 11,78,475 deaths globally, since late December 2019, when WHO was notified about an unusual outbreak of pneumonia in Wuhan, China, until October 30, 2020 (WHO, 2020a).

In Europe, the first cases of infections caused by the newly identified virus, SARS-CoV-2, were recorded on January 25, 2020 (WHO, 2020b) and spread rapidly; 11,062,715 cases and 285,135 deaths were reported by October 31, 2020 (WHO, 2020c).

In Romania, the first case was reported on February 26, 2020 (Rapid analysis of Romanian COVID-19 confirmed cases, 2020), 241,339 cases and 6,968 deaths being confirmed by the end of October 2020 (WHO, 2020c).

Despite the large amount of available data regarding adult patients with SARS-CoV-2 infection, there are still limited data regarding SARS-CoV-2 infection in children. From what we know so far, compared with adults, COVID-19 in children is less frequent and less severe. There is a low prevalence of diagnosed children with SARS-CoV-2 all over the world. In United States (Bialek et al., 2020), less than 2% of the cases were reported in children, similar to the reports from Spain (Tagarro et al., 2020) or Italy (Livingston & Bucher, 2020). Other countries, with higher rates of community testing, found that only 4% of the cases were children (Williams et al., 2020).

In Romania, the pediatric population accounted for 6.55% (15,817 cases) of all the reported cases at the national level. 5,165 out of 15,817 cases (32.62%) were confirmed in children up to 9 years old and 10,657 (67.38%) were confirmed in children between 10–18 years old (Sava, 2020a).

SARS-CoV-2 infection in children is also less severe than in adults, including patients traditionally considered to be at high risk of severe infections (Boulad et al., 2020). Although SARS-CoV-2 infection in most children is commonly asymptomatic or with mild symptoms, in some cases pediatric patients can develop severe disease, complications and require prolonged intensive care support (Lu et al., 2020; Riphagen et al., 2020). Moreover, a severe hyperinflammatory condition, called multisystem inflammatory syndrome (MIS-C), was described in children and adolescents. This syndrome that was first described in Europe in April, 2020, usually occurs 2–6 weeks after acute SARS-CoV-2 infection and can affect multiple organ systems (cardiac, gastrointestinal, hematological, dermatological, neurological, respiratory, renal systems) (Abrams et al., 2021).

Because the information regarding COVID-19 in pediatric patients is still sparse, we consider that it is important to analyze the characteristics of the children diagnosed with SARS-CoV-2 infection, confirmed in our hospital. Such an analysis is helpful in more than one direction: to obtain a more accurate picture of the disease spectrum in this specific population, to investigate different factors of prediction for a probable unfavorable evolution in specific groups, thus allowing to monitor and treat them promptly, to early diagnose any potential infected child which would allow to implement specific measure for transmission control.

To our knowledge, this is the first Romanian report that describes the characteristics of COVID-19 in pediatric patients.

### Objectives

The purpose of this paper is to analyze the epidemiological and clinical characteristics of the children presented to “Marie Curie” Emergency Children’s Hospital, Bucharest, Romania who were diagnosed with SARS-CoV-2 infection. Due to the fact that information on pediatric patients is still limited, we aim to highlight the epidemiological and clinical data regarding SARS-CoV-2 infection in children and adolescents, to improve the understanding of the disease in this age group and inform physicians during the ongoing COVID-19 pandemic.

## Materials & methods

We conducted a retrospective, observational study, in “Marie Curie” Emergency Children’s Hospital, a tertiary, multidisciplinary hospital from Bucharest, Romania, regarding the characteristics of the pediatric patients diagnosed with SARS-CoV-2 infection during a seven months period (April 1^st^, 2020–October 31^st^, 2020). The study was approved by the Ethics Committee of our hospital (SPIAAM no. 39945/29.10.2020) and we received written informed consent from participants in our study.

Between April 1^st^, 2020-October 31^st^, 2020, patients aged 0–18 years who presented to “Marie Curie” Emergency Children’s Hospital, Bucharest, Romania were tested for SARS-CoV-2 infection, using real-time reverse transcription polymerase chain reaction (RT-PCR). The samples of nasopharyngeal swabs were collected in our hospital and RT-PCR was performed in external laboratories.

According to the hospital protocol, all the patients who were admitted, both with suspicion of SARS-CoV-2 infection and those without suspicion but who had other non-COVID-19 associated health problems for which they sought medical treatment, were tested for SARS-CoV-2 infection. SARS-CoV-2 infection suspected patients who needed inpatient care were isolated in designated wards and, after the tests results were obtained, they were either relocated in the other wards from the hospital (those with negative results) or they were transferred in COVID-19 designated hospitals (those with positive results). The patients with symptoms compatible with SARS-CoV-2 infection, who presented to the emergency room, but who didn’t have criteria for inpatient treatment, were also tested and were given indication to remain at home until the test result was available; those with positive results were informed and given further recommendations according to the national regulations. The non-suspect patients who were admitted for other health problems, were also passively screened for SARS-CoV-2 and those who were found positive, were transferred in COVID-19 hospitals.

The suspicion of SARS-CoV-2 infection was established based on National Methodology of Surveillance for the Acute Respiratory Syndrome with the novel coronavirus (COVID-19), which was available from march 2020 and updated regularly (CNSCBT, 2020).

The suspect cases were evaluated for SARS-CoV-2 infection, according to the methodology that was in place at the time of their presentation. Over the last 7 months, the definition of the suspect case was updated and recommendations for testing prioritization were established. Initially, the suspect case was defined as: (1) Patient with acute respiratory infection (sudden onset of at least one of the symptoms: cough, fever, shortness of breath without other explanation) and history of travel/stay in a country/region with community transmission in the last 14 days prior to the onset of the symptoms; (2) Patient with acute respiratory infection and who came in close contact with a COVID-19 confirmed case in the last 14 days prior to the onset of the symptoms; (3) Patient with severe acute respiratory infection (fever and cough and shortness of breath) without other explanation for these symptoms and who needs inpatient care. The present definition of the suspect case includes: (1) Any person with sudden onset of fever and cough or (2) Any person with sudden onset of at least three symptoms from: fever, cough, fatigue, headache, myalgia, odynophagia, rhinorrhea, shortness of breath, lack of appetite/nausea/vomiting, diarrhea, altered mental status, recent onset of anosmia or ageusia, in the absence of other explanation, or (3) Any person with pneumonia/bronchopneumonia with or without pleural effusion, or (4) Patient with severe acute respiratory infection (fever and cough and shortness of breath) without other explanation for his symptoms and who needs inpatient care; (5) For children younger than 16 years, SARS-CoV-2 infection can be suspected in case of digestive symptoms (vomiting, diarrhea) that are not associated with their diet.

The diagnosis of SARS-CoV-2 infection was confirmed based on National Methodology of Surveillance for the Acute Respiratory Syndrome with the novel coronavirus (COVID-19): positive RT-PCR, regardless of the presence of clinical signs and symptoms (CNSCBT, 2020).

Other investigations were also performed according to the clinical picture of the patient and to the recommendation of the attending physician: chest X-ray, hematological, C reactive protein, procalcitonine, liver enzymes, kidney panel.

The disease severity was defined, according to the national regulations of the Romanian Ministry of Health, as follows: asymptomatic (patient with positive PCR-SARS-CoV-2 test and with no signs or symptoms of disease); mild (uncomplicated upper respiratory tract viral infection (e.g., fever, cough, sore throat, malaise, headache, muscle pain) without shortness of breath, dyspnea, or abnormal chest imaging); moderate (clinical and radiological signs of non-severe pneumonia and SaO2 > 90% in room air), severe (e.g., severe pneumonia, hypoxia, dyspnea, tachypnea requiring hospitalization); and critical (e.g., severe pneumonia, acute respiratory distress syndrome, septic shock, and/or multiple organ dysfunction requiring hospitalization in intensive care).

The hospital’s Department for Prevention of Healthcare Associated Infections recorded all the epidemiologic and clinical data of the SARS-CoV-2 positive patients. We retrospectively analyzed the data from the epidemiological survey records, including: demographics, medical history and underlying diseases, epidemiological history, laboratory tests, radiological findings.

### Statistical analysis

Descriptive statistics were determined using SPSS software (version 25, IBM). Statistical analysis of the data was performed to compare variables, as appropriate. Categorical variables are presented as number and frequency rate. Continuous variables are presented as median and interquartile range. Nonparametric tests were applied to compare independent samples. Nonparametric tests for independent samples were used to compare the medians of the two groups of patients regarding the median values of leukocytes, lymphocytes, neutrophils, platelets and C reactive protein. The statistical significance was assessed by calculating statistical significance threshold (*p* value). *p* was considered significant for value less than 0.05.

## Results

### Characteristics of the patients

Patients’ characteristics are presented in Table 1.

**Table 1:** Characteristics of the patients.

Parameter	Indicators	Total *N* = 172	With symptoms *N* = 142 (85.56%)	Without symptoms *N* = 30 (17.44%)	*p*
Gender	Female	79 (45.93%)	65 (45.78%)	14 (46.66%)	1
	Male	93 (54.07%)	77 (54.22%)	16 (53.33%)	1
Age distribution		15 days–18 years (median 6.35)	15 days–18 years (median 7)	5 months–18 years (median 6.15)	0.84
	0–1 years	31 (18.02%)	28 (19.72%)	3 (10%)	0.29
	1–4 years	41 (23.84%)	31 (21.83%)	10 (33.33%)	0.23
	5–9 years	32 (18.6%)	27 (19.01%)	5 (16.67%)	1
	10–14 years	29 (16.86%)	25 (17.61%)	4 (13.33%)	0.78
	15–18 years	39 (22.67%)	31 (21.83%)	8 (26.67%)	0.63
Exposure to identified source		47 (27.32%)	41 (28.87%)	6 (20%)	0.2
	Family	37 (78.72%)	32 (78.05%)	5 (83.33%)	1
	One family member	35	30	5	0.62
	Two family members	1	1	0	1
	Three family members	1	1	0	1
	Community	8 (17.02%)	8 (19.51%)	0 (0%)	0.57
	Institution of care	1 (2.13%)	1 (2.44%)	0 (0%)	1
	During transport to hemodialysis	1 (2.13%)	0 (0%)	1 (16.66%)	0.12
Underlying medical conditions		28 (16.28%)	14 (9.86%)	14 (46.66%)	0.00001
	Obesity	2 (1.16%)	2 (14.29%)	0 (0%)	0.48
	Neurologic	8 (28.57%)	5 (35.71%)	3 (28.54%)	0.1
	Oncologic/hematologic	7 (25%)	1 (7.14%)	6 (42.68%)	0.0001
	Nephrologic	6 (21.23%)	4 (28.54%)	2 (14.28%)	0.28
	Diabetes mellitus type 1	3 (10.71%)	1 (7.14%)	2 (14.28%)	0.07
	Respiratory	2 (7.14%)	2 (14.28%)	0 (0%)	1
	Chronic orthopaedic condition	2 (7.14%)	0 (0%)	2 (12.28)	0.02
Number of underlying conditions	1	23 (82.14%)	11 (78.57%)	12 (85.71%)	1
	2	4 (14.29%)	2 (14.29%)	2 (14.29%)	0.14
	4	1 (3.57%)	1 (7.14%)	0 (0%)	1

**Note:**

Nonparametric tests were applied to compare independent samples *p* < 0.05.

Between April 1, 2020–October 31, 2020, 19,619 patients aged 0–18 years were seen in our hospital. RT-PCR for SARS-CoV-2 was performed in 7,902 (40.28%).

A total of 172 patients were confirmed with SARS-CoV-2 infection, representing 2.18% of the total 7,902 RT-PCR tests.

A total of 79 (45.93%) female patients and 93 (54.07%) male patients were identified, with sex ratio males per females of 1.18.

The number of the patients with positive RT-PCR-SARS-CoV-2 tests increased gradually from month to month, throughout the studied time frame: three patients (1.74%) in April, four patients in May (2.32%), 10 patients in June (5.81%), 21 patients (12.21%) in July, 31 patients in August (18.02%), 39 patients (22.67%) in September and 64 patients (37.21%) in October.

Median age was 6.35 year (15 days–18 years). The distribution by age groups is detailed in Table 1.

In 47 cases (27.32%) we found a history of exposure to an identified source of infection: 37 patients (78.72%) were exposed to a source from their family, eight patients (17.02%) had exposure in the community, one patient (2.13%) was in institutionalized care where a cluster of SARS-Cov-2 was identified and one patient (2.13%) was transported to our hospital for hemodialysis and he traveled in the same ambulance with a SARS-CoV-2 positive patient. Regarding the patients with family exposure, at the time of the epidemiological inquiry, in 35 cases we identified exposure to 1 family member (mother or father in 30 cases, brother in one case, grandmother in two cases and for two brothers, the source of the infection was their aunt), in one case the child had been exposed to both his SARS-CoV-2 confirmed parents and 1 child had exposure to 3 family members (mother, father and grandfather). Out of the 172 patients, three pairs of two brothers came from the same families. Among the 47 patients with identified source, 6 patients didn’t have any symptoms compatible with SARS-CoV-2 infection.

In 28 (16.28%) patients we identified at least one previous medical condition: obesity (two patients), neurological or neurosurgical conditions (i.e., epilepsy, congenital hydrocephalus–8 patients), oncological or hematological conditions (i.e., acute or chronic lymphocytic leukemia, neuroblastoma–7 patients), nephrological conditions including chronic kidney disease (six patients), diabetes mellitus type one (three patients), cystic fibrosis (one patient), asthma (one patient), chronic orthopedic conditions (two patients). Most of the patients had only underlying condition; however, in five cases we identified more than one previous medical conditions.

### Clinical characteristics

Out of 172 SARS-CoV-2 positive patients, 30 (17.44%) had no symptoms and 142 (85.56%) were symptomatic. Clinical findings on presentation included at least one of: fever (112 patients; 78.87%), cough (57 patients; 40.14%), nasal congestion and rhinorrhea (34 patients; 23.94%), headache (26 patients; 18.31%), odynophagia and dysphagia (26 patients; 18.31%), nausea (14 patients; 9.86%), vomiting (21 patients; 14.79%), diarrhea (21 patients; 14.79%), abdominal pain (15 patients; 10.56%), myalgia (19 patients; 13.38%), anosmia/ageuzia (15 patients; 10.56%), neurological symptoms (11 patients; 7.75%) such as: irritability and restlessness (3 patients, one of them being a patient with a respiratory comorbidity), hypotonia (in a 12 years old female patient who was previously diagnosed with benign congenital hypotonia), generalized febrile seizures (one patient), pronounced drowsiness (two patients), dizziness (three patients), balance disorder (one patient), arthalgia (five patients; 3.52%), fatigue (five patients; 3.52%), apneic event (two patients; 1.41%) and nonspecific rash (one patient; 0.7%). None of the patients had hypoxia or dyspnea.

The comparison between the symptomatic patients with and without underlying conditions did not show any statistically significant differences between the two groups regarding any specific type of symptom (Table 2).

**Table 2:** Comparison of the symptoms between the group of patients with and without underlying conditions.

Symptoms	Symptoms in patients with underlying conditions (*n* = 14 patients)	Symptoms in patients without underlying conditions (*n* = 128 patients)	*p*
Fever*	10 (71.43%)	102 (79.68%)	0.49
Cough	4 (28.57%)	53 (41.40%)	0.40
Nasal congestion and rhinorrhea	2 (14.29%)	32 (25%)	0.36
Headache	3 (31.43%)	23 (17.97%)	0.72
Odynophagia and dysphagia	1 (7.15%)	25 (19.53%)	0.46
Digestive symptoms^#^	5 (35.71%)	66 (51.56%)	0.18
Myalgia	2 (14.29%)	16 (12.5%)	0.69
Anosmia/ageuzia	2 (14.29%)	13 (10.15%)	0.64
Neurological symptoms^◊^	2 (14.29%)	9 (7.03%)	0.29
Artharlgia	2 (14.29%)	3 (2.34%)	0.07
Fatigue	2 (14.29%)	4 (3.12%)	0.1
Apneic event	0 (0%)	2 (1.56%)	1
Nonspecific rash	0 (0%)	1 (0.78%)	1

**Notes:**

* Fever ≥ 38 °C.

# digestive symptoms: nausea, vomiting, diarrhea, abdominal pain.

◊ neurological symptoms: restlessness and irritability, dizziness, drowsiness, febrile seizures, generalized hypotonia, balance disorder; Nonparametric tests were applied to compare independent samples *p* < 0.05.

Most of the symptomatic patients had three symptoms (52 patients; 36.62%); 36 patients (25.35%) had two symptoms; 23 patients (16.2%) had four symptoms; 21 patients (14.8%) had only one symptom; nine patients (6.33%) had five symptoms and in one patient (0.7%) were identified six symptoms.

According to the patients’ symptoms, we could identify three main groups of clinical phenotypes: a cluster of respiratory symptoms which included fever, cough, rhinorrhea, nasal congestion, a cluster of systemic enteric illness which included headache, myalgia, sore throat, vomiting, abdominal pain, diarrhea, fatigue, rash and a cluster of neurological symptoms which included irritability and restlessness, hypotonia, generalized febrile seizures, drowsiness, dizziness, balance disorder. Systemic enteric illness was the most frequent (48 patients, 33.8%), followed by respiratory cluster (32 patients, 22.54%) and neurological cluster was extremely rare (three patients, 2.11%). Fever alone was present in 18 patients (12.68%) and in 41 cases (28.87%) we found an overlap of the clusters: respiratory and systemic enteric illness (27 patients, 19.01%), respiratory and neurological symptoms (four patients, 2.82%), neurological and systemic enteric illness (two patients, 1.4%) or all three clusters (eight patients, 5.63%). We analyzed the distribution of the clinical clusters within the two groups of patients, with and without comorbidities. Patients with comorbidities developed fever alone (three patients), systemic enteric illness (5 patients), respiratory illness (three patients) or an association of respiratory, neurological and systemic enteric illness (three patients). Patients without comorbidities presented fever alone (15 patients), systemic enteric illness (43 patients), respiratory illness (29 patients), neurological symptoms (three patients) or an association of respiratory and systemic enteric illness (27 patients), neurological and systemic enteric illness (two patients) or respiratory, neurological and systemic enteric illness (five patients). When we compared the two groups regarding significant differences from the clinical phenotypes point of view, we found that patients with comorbidities have a higher chance for developing an overlap of symptoms from all the clinical clusters (*p* = 0.03).

### Laboratory and radiological findings

Laboratory and radiological examination were not performed in all the patients. In Table 3 are presented the data collected from the patients who underwent these evaluations.

**Table 3:** Laboratory and radiological characteristics.

Parameter	Indicators	Total *N* = 172	With symptoms *N* = 142 (85.56%)	Without symptoms *N* = 30 (17.44%)	*p*
Chest X-ray		50 (29.07%)	47 (33.1%)	3 (10%)	
	Normal	25 (50%)	22 (46.88%)	3 (100%)	0.57
	Bilateral peri and infrahilar mildly increased interstitial marking	22 (44%)	22 (46.88%)	0 (0%)	0.01
	Unilateral alveolo-interstitial pneumonia	3 (6%)	3 (6.38%)	0 (0%)	1
Complete blood count		131 (76.16%)	110 (77.46%)	21 (70%)	
	Leukocytes (4.5–10 × 10^9^ cells/L)	6,860 (1,770–103,000)	6,910 (4,600–103,000)	6710 (1,760–14,640)	0.661
	Lymphocytes (1.5–6.5 × 10^9^ cells/L)	2180 (260–11,640)	2190 (460–11,640)	1710 (260–84,600	0.97
	Neutrophils (1.8–8 × 10^9^ cells/L)	3110 (390–88,870)	3100 (390–88,870)	3190 (930–12,050)	0.97
	Monocytes (0–1 × 10^9^ cells/L)	850 (100–5700)	900 (260–5,700)	440 (100–2,860)	0.019
	Platelets (150–450 × 10^9^ cells/L)	252,000 (37,000–637,000)	254,000 (124,000–637,000)	252,000 (27,000–511,000)	0.97
C reactive protein (<5 mg/l)		3.62 (0.02–305.2)	3.77 (0.02–305.2)	2.72 (0.26–71.88)	1

**Note:**

Nonparametric tests were applied to compare independent samples *p* < 0.05.

Laboratory workup was performed according to the medical decision of the attending physician.

Complete blood count was evaluated for 131 patients (76.16%). Leukopenia was identified in 20 patients (15.27%) and leukocytosis in 24 patients (18.32%). However, four of the patients with elevated leukocytes had oncological or surgical pathologies which might have explained the hematological changes. Lymphopenia was noted in 34 cases (25.95%), and in 11 cases (8.4%) an elevated number of lymphocytes was observed. 20 patients (15.27%) had neutrophilia and 16 patients (12.21%) had decreased neutrophils count. Thrombocytopenia was identified in two patients (1.53%), one of the patients having hematological underlying conditions as an explanation.

C-reactive protein was performed for 114 patients, in 46 (35.11%) of them an elevated value being noticed.

Other tests (procalcitonine, aspartate aminotransferase and alanine aminotransferase, kidney function) were performed in a very low proportion of patients and could not be analyzed in order to draw any significant conclusion.

Chest X-ray was performed on initial evaluation for 50 patients (29.07%). In half of them, there were no changes, while in 22 cases (44%) showed peri and infrahilar mildly increased interstitial markings and in three cases (6%) revealed alveolo-interstitial pneumonia (Table 3). Out of the 50 performed chest X-rays, 47 (94%) were done for patients with symptoms: in seven out of 14 patients with comorbidities (four were abnormal and three were normal) and in 40 out 128 patients without comorbidities (21 were abnormal and 19 were normal). There was no statistical difference between patients with or without comorbidities regarding radiological changes (*p* = 1). In asymptomatic patients, chest X-ray was performed and found to be normal for two patients with and one patient without underlying diseases.

### Management

Due to the fact that our hospital was not a COVID-19 hospital and according to the national regulations, all the patients admitted to our hospital, who were diagnosed with SARS-CoV-2 infection and didn’t have a pathology that required further inpatient treatment in our hospital, were transferred for isolation, evaluation and management, to one of the COVID-19 designated hospitals that have pediatric wards. This was the case for all the hospitalized patients evaluated in this study.

A total of 68 (39.53%) patients (24 asymptomatic and 44 symptomatic) patients were admitted to the hospital, requiring treatment for other medical condition (for asymptomatic patients) or inpatient care for their symptoms (for symptomatic patients). The admitted patients awaiting for the tests results were monitored and treated according to their symptoms and non-COVID-19 pathologies, none of them requiring escalation of the treatment. A total of 15 out of 24 asymptomatic patients confirmed with SARS-CoV-2 infection were transferred once the condition for which they were admitted was resolved; 11 of them were children with comorbidities. A total of nine out of 24 asymptomatic patients were discharged and isolated at home. A total of 35 out of 44 symptomatic patients were transferred once the test result was available and nine were discharged before, due to the favorable evolution. A total of 98 symptomatic patients, who were evaluated and tested for SARS-CoV-2 infection in the emergency room and who did not require inpatient treatment, were discharged at home, with symptomatic treatment and isolation recommendations. All the patients who were discharged at home, once the test result was available, were notified and given further instructions according to the local regulations for COVID-19 patients.

## Discussion

To our knowledge, this is the first report on pediatric COVID-19 patients in Romania, with data from children and adolescents who were seen or managed within a tertiary health-care institution.

Since the beginning of the SARS-CoV-2 pandemic, it was noted that the disease was less frequent in children and also that among the infected children, a vast majority is spared of severe complications of COVID-19. In our study, SARS-CoV-2 infection was confirmed in 2.18% of the pediatric patients who presented to our hospital, during a seven months period. A review by the Chinese Center for Disease Control (CDC) showed that less than 1% of cases occurred in children younger than 10 years and most of them had non-severe disease (Wu & McGoogan, 2020). In Italy, pediatric patients accounted for 1.8% of the total infections (Bellino et al., 2020), consistent with data from Spain (Tagarro et al., 2020). The same low rate was initially reported in United States, with only 2% of the cases being reported in pediatric patients in April 2020 (Bialek et al., 2020). However, as the time passed and United States became one of the nations with extremely high rates of infection, the rates of infected children increased. According to a joint report from the American Academy of Pediatrics (2020) and the Children’s Hospital Association (CHA), pediatric cases have risen from 2% of cumulative reported cases in April to 10.5% in September, 2020 (Sisk et al., 2020; American Academy of Pediatrics, 2020).

In our study, an uptrend of monthly confirmed cases was noticed. At the beginning of the pandemics, the lockdown measures controlled the spread of the disease among all age groups, including children and adolescents. As time has passed, and the measures were lowered, especially during the summer time breaks, we witnessed an increasing number of cases. Although we don’t have clear public information in this respect, we might hypothesize that the beginning of the school year could be another explanation. We may speculate that, in the context of the rapid spread and the increasing rates of SARS-CoV-2 infections, we will also see more and more cases in children, as the pandemic is ongoing. An interesting observation was made in respect to the age distribution of the patients from our study compared to the national data. In our study, children aged 0–9 years represented 104 (60.47% of the study population), whereas children aged 10–18 years represented 68 (39.53%). Conversely, at national level, children aged 0–9 years represented 31.69% of the total pediatric cases and children aged 10–18 years represented 68.31% (Sava, 2020b). This inverted proportion of age groups between cases reported at national level (both hospitalized and non-hospitalized cases) and cases from our hospital, may be due to the fact that although overall older children and adolescents are more frequently affected, parents of younger children seek medical help more often than those of older children. *Also*, children aged 1–4 years have closer contacts within children’s collectivities and facial mask is not mandatory for this age group and even if it would be, small children could not comply with this rule, therefore being more exposed. Interestingly, 18.02% of the cases were in children aged 0–1 year, who probably were infected from their households and other caregivers. The transmission of SARS-CoV-2 infection from adults to children has been confirmed and attributed to high prevalence of the disease in children. Exposure to an identified source was identified in many SARS-CoV-2 infected children, in up to 90% of the evaluated cases in some studies (Zhang et al., 2020; Cao et al., 2020). In our study, a source of infection was identified in over a quarter of the cases, mostly in the household environment, children being infected by adult relatives, which may explain the number of cases identified in small children. We anticipate that with the increasing number of infected adult contacts, the number of pediatric infections will also increase concomitantly and more family clusters will be identified in the future.

Besides the low prevalence of the disease in children, the available data highlight the fact that most children exhibit mild, if any, illness caused by SARS-CoV-2. In our study, 17.44% of the patients with positive RT-PCR didn’t have either symptoms, or radiological features of disease. Our observation is consistent with reports by other authors (Lu et al., 2020). A multicentre cohort study involving 82 participating health-care institutions across 25 European countries showed that 16% of the SARS-CoV-2 infected children were asymptomatic (Götzinger et al., 2020). Traditionally, patients with underlying conditions are considered high risk for severe infections. Most of severe and critical cases or deaths in COVID-19 children occurred in those with comorbidities, the same as in adult patients (Ludvigsson, 2020). Lu et al. (2020), in a study including 171 patients, reported 3 children with hydronephrosis, leukemia and intussusception, who required mechanical ventilation (Lu et al., 2020). Other authors also found a significantly increased risk of severe COVID-19 and COVID-19-associated mortality in children with comorbidities. A systematic review and meta-analysis by Tsankov et al. found that 5.1% of children with comorbidities presented severe COVID-19 compared to 0.2% of children without comorbidities and COVID-19-associated mortality was 1.5% in children with pre-existing conditions and 0.03% in children without comorbidities (Tsankov et al., 2021). Swann et al. (2020) noted that children with comorbidities were more likely to be admitted to critical care than those without comorbidities and comorbidities most commonly associated with critical care admission were prematurity, respiratory and cardiac comorbidities. Six patients died in the hospital, all of them being patients with severe comorbidities or illnesses (Swann et al., 2020).

In our study, 28 (16.28%) of the children infected with SARS-CoV-2 had underlying conditions and five of them had more than one comorbidity. Interestingly, in our study, the rate of comorbidities in asymptomatic patients was significantly higher than in symptomatic patients. In the same manner, we found a higher rate of patients with oncohematological comorbidities than in symptomatic group. This is consistent with other studies that noted that children with certain underlying conditions, such as cancer, who are infected with SARS-CoV-2 are not more vulnerable than other children and do not usually develop severe illness (Boulad et al., 2020; Choi et al., 2020). Other authors have also seen a low risk of severe COVID-19 in children with comorbidities. Brisca et al. (2021) focused on the evolution of 37 pediatric patients with comorbidities and COVID-19 and concluded that although 78% were hospitalized, 81% had minimal or absent respiratory symptoms, only two required oxygen support and none of them needed mechanical ventilation or intensive care admission (Brisca et al., 2021).

In symptomatic COVID-19 children, the most common described symptoms are fever, cough, gastrointestinal symptoms; headache and myalgia are also reported (Williams et al., 2020; Götzinger et al., 2020; Hoang et al., 2020). In our study, the most frequent symptom was pyrexia (temperature above 38 °C), followed by digestive symptoms (nausea, vomiting, diarrhea, abdominal pain), cough, nasal congestion and rhinorrhea, headache, odynophagia and dysphagia, myalgia. Anosmia, with or without ageusia were also recorded in 10% of our patients, which is lower than the rates described in adults or reported in children by other authors (Qiu et al., 2020). As it has been suggested, these dysfunctions may represent early or the only symptom of SARS-CoV-2 infection in both adults and children and they may help to identify oligosymptomatic or atypically symptomatic patients. However, these symptoms may be of little help, especially in small children, who might be unable to describe them. In our study, anosmia and ageusia were recognized by children above 6 years of age. Other symptoms were present in a smaller number of cases: other non-specific and non-severe neurological symptoms (restlessness and irritability, dizziness, drowsiness, febrile seizures, generalized hypotonia, balance disorder), arthralgia, fatigue, apneic event, nonspecific rash.

When we compared patients with underlying medical conditions with those without underlying medical conditions, in terms of any particular symptom that might be present in the first group, we didn’t identify any statistically significant differences for any of the symptoms analyzed separately. Swann et al. (2020) pointed out three distinct clusters of clinical phenotypes: the most frequent was a cluster of systemic mucocutaneous-enteric illness (headache, myalgia, sore throat, vomiting, abdominal pain, diarrhea, fatigue, rash, lymphadenopathy, and conjunctivitis) followed by a cluster of upper and lower respiratory symptoms (cough, fever, shortness of breath, runny nose, lower chest wall indrawing, wheeze), and a rarer cluster of neurological symptoms (seizures, confusion) (Swann et al., 2020). We could also identify these clusters of symptoms in our patients, but with some differences regarding the symptoms from each cluster. Respiratory symptoms included fever, cough, rhinorrhea, nasal congestion; we didn’t identify lymphadenopathy and conjunctivitis in the second cluster; we included more symptoms in the neurological cluster (irritability and restlessness, hypotonia, generalized febrile seizures, drowsiness, dizziness, balance disorder). In our study, systemic enteric illness was also the most frequent, followed by respiratory cluster and neurological cluster was extremely rare. We also found that patients with comorbidities have a higher chance for developing an overlap of symptoms from all the clinical clusters.

From the experience with COVID-19 adults, we have learnt that an elevated number of leukocytes and neutrophils, as well as a low lymphocytes count are associated with a severe evolution (Lippi & Plebani, 2020). Unlike adults, in children with COVID-19, a normal white blood cells count is usually present and lymphopenia is rarer, which may explain the milder form of disease in pediatric population (Liao et al., 2002; Castagnoli et al., 2020). When abnormalities of the leukocytes count were found, leukopenia was the most common (Patel, 2020; Meena et al., 2020). A low platelets count has been more frequent identified in critically ill children and has been associated with respiratory deterioration in children (Romani et al., 2020; Bhumbra et al., 2020). The findings in our study showed the same: most of the patients had a normal hematological profile and in the few cases in which “classical” markers of a possible unfavorable evolution where noted, they were not corelated with a more severe evolution. Also, when we compared the complete blood count for symptomatic against asymptomatic patients, we didn’t find any significant differences, except for the monocytes count. Xiong et al. (2020) found that symptomatic COVID-19 infected children had a significantly higher monocytes count than asymptomatic patients (Xiong et al., 2020). Regarding this aspect, there aren’t homogenous conclusions at this time. There are studies in adults that concluded that monocytes count was usually in normal range in COVID-19 patients, however it could be towards the lower range in severe patients (Cong-Ying et al., 2020). In other studies, no differences were found between non-severe and severe patients in terms of monocytes count (Qin et al., 2020), whilst other authors showed that COVID-19 patients had a higher monocytes count compared to healthy individuals, but still within the normal range (Kermali et al., 2020).

Although imaging studies of children with known or suspected SARS-CoV-2 infection are not routinely recommended, they play an important role especially in pediatric patients with a moderate to severe COVID-19 in order to establish a baseline, to assess any complications related to specific underlying conditions, to monitor the disease progression or to evaluate the treatment response (American College of Radiology, 2020; Foust et al., 2020). Chest imaging findings in children with COVID-19 are frequently normal or mild. However, studies are describing heterogenous aspects of imaging studies in SARS-CoV-2 infected children. Lower lobes are commonly affected, but the upper or middle lobe involvement, as well as diffuse or multifocal disease is described. Although the most common described pattern was ground-glass opacity, patchy consolidations or a halo sign of ground-glass opacification around areas of consolidation were also found (Shelmerdine et al., 2020).

In our study, 50 patients (29.07%) underwent Chest X-ray on initial evaluation. We found that half of the symptomatic patients had signs of non-severe pneumonia. None of them had the classical ground-glass pattern. Chest X-ray was also evaluated in three asymptomatic children, and it was unremarkable, although even in asymptomatic patients’ radiological findings might be found (Chan et al., 2020). We found no statistical difference between patients with or without comorbidities regarding radiological changes.

Based on the presence or absence of the symptoms, the amplitude of the symptoms and radiological signs, we could conclude that patients who presented to our hospital for evaluation and management, exhibited either asymptomatic infection (17.44%), mild disease (68.02%), or moderate disease (1.04%). These findings mirror one particularity of the global pandemic caused by SARS-CoV-2, namely that unlike many respiratory viruses, pediatric patients have been relatively spared by severe illness. The explanation might come from several directions: fewer underlying conditions, the differences in their immune response to the virus and lower predisposition to proinflammatory states, differential expression of ACE2 in this group of patients, which may attenuate viral entry, ongoing viral replication and subsequent inflammation, hypoxia and tissue injury (Williams et al., 2020).

In our study, about 60% of the patients didn’t require inpatient treatment and almost 40% of the patients were admitted. About one third of the admitted patients were asymptomatic, being admitted for the treatment of another underlying condition, either acute or chronic; they underwent the screening imposed by our internal regulations and tested positive for SARS-CoV-2 infection. About two thirds of the patients were symptomatic. None of the admitted patients required admission to the ICU. According to our national regulations, once the SARS-CoV-2 infection was confirmed, the patients were transferred to COVID-19 designated hospitals. To our knowledge, none of these patients had a further unfavorable or complicated evolution, including those with predisposing factors for severe infections. Our findings are concurrent with other authors, as severe acute phase of infection requiring admission to ICU is rare in children. At Wuhan Children’s Hospital, only 1.8% out of 171 COVID-19 children required ICU admission (Lu et al., 2020). Tagarro et al. (2020) found that although a high proportion of children required hospitalization (61%), only 9.8% of the them required admission to ICU (Tagarro et al., 2020). In North America, most of SARS-CoV-2 infections in children, did not required hospitalization and those requiring ICU admission frequently had complex underlying conditions that weighted towards the need of ICU (Pathak et al., 2020; Andre et al., 2020; Garg et al., 2020). The 25 European countries multicenter cohort study regarding COVID-19 in children and adolescents, highlighted that 62% of the patients were admitted to hospital, 8% of them required ICU admission and 4% needed mechanical ventilation (Götzinger et al., 2020).

There are limitations of this study. Firstly, the recorded data were non-homogenous (not all the patients underwent chest-Xray, a unitary laboratory tests panel was not applied), the laboratory and imaging tests were ordered and performed according to the clinical judgment of the attending physician, without involvement from the study team, which only recorded the available data. Therefore, a decision was made not to collect detailed data on all laboratory parameters when they were available in a very small number. Also, we consider that the very different sizes of the two compared groups (with and without underlying conditions) represented an obstacle for drawing more significant clinical conclusions. Another limitation regards the fact that we could not directly evaluate the short and especially the long-term evolution of the patients, which remains to be seen. In the future, we consider this to be an important step in the management of children affected by SARS-CoV-2 infection. In contrast with adults, were there are preliminary concerning data regarding the longer-term respiratory, cardiac and neurological sequelae, in children, aside the sequelae from the multisystem inflammatory syndrome associated with SARS-CoV-2, there are still little outcome data beyond acute infection.

These days we are experiencing a rising number of cases in Romania, as well as in many other countries. Therefore, we are expecting to see an increasing rate of SARS-CoV-2 infection in children also, and it is important to keep monitoring the characteristics of the disease, its evolution, possible complications and sequelae in the pediatric patients, because with the pandemic progression, the clinical picture in children might change in the future.

## Conclusions

Our results confirm less severe SARS-CoV-2 infection in pediatric patients than in adults. The results from our study showed that patients who presented to our hospital exhibited asymptomatic, mild and moderate illness due to infection with SARS-CoV-2, including the group of children with who are classically considered high risk patients for severe disease, such as children with cancer, cystic fibrosis, immunosuppression and other important comorbidities.

## Supplemental Information

10.7717/peerj.11560/supp-1 Supplemental Information 1 Raw data. Characteristics of the patients, comparison of the symptoms between the group of patients with and without underlying conditions and laboratory and radiological characteristics.

## Additional Information and Declarations

### Data Availability

The following information was supplied regarding data availability: The raw measurements are available in the Supplemental File.
